# Assessment of Coronavirus Disease 2019 Community Containment Strategies in Shenzhen, China

**DOI:** 10.1001/jamanetworkopen.2020.12934

**Published:** 2020-06-22

**Authors:** Xiao-Ming Zhang, Hui-Er Zhou, Wen-Wu Zhang, Qing-Li Dou, Ye Li, Jian Wei, Rui Hu, Jiangping Liu, Andy S. K. Cheng

**Affiliations:** 1 Department of Emergency, Affiliated Baoan Hospital of Southern Medical University, People’s Hospital of Shenzhen, Baoan District, Shenzhen, China; 2 Department of Rehabilitation Sciences, Hong Kong Polytechnic University, Hong Kong, China

## Abstract

**Importance:**

A new outbreak of pneumonia caused by severe acute respiratory syndrome coronavirus 2 in Wuhan, China, is spreading rapidly around the globe. Limited information on control in community settings is available.

**Objective:**

To detail measures enacted within a community to prevent the spread of coronavirus disease 2019 (COVID-19) and to evaluate the spread of COVID-19 associated with implementation of the program.

**Design, Setting, and Participants:**

This case series study details the implementation of prevention measures in a specific community setting among community-dwelling individuals exposed to or at risk of COVID-19 in Haiyu, Shenzhen, China, from January 23 to April 10, 2020.

**Exposures:**

Community containment strategies for tracking, quarantine, and management were strictly, cooperatively, and effectively implemented by a team that included a general practitioner, a community manager, and public safety bureau officials.

**Main Outcomes and Measures:**

Number of locally acquired cases with indirect links to confirmed COVID-19 cases. Diagnosis with COVID-19 was confirmed when throat swab samples tested positive for severe acute respiratory syndrome coronavirus 2 on reverse transcription–polymerase chain reaction.

**Results:**

Approximately 34 686 individuals live in Haiyu, including 2382 residents aged 65 years or older. Seven individuals with COVID-19 acquired outside the community (age, 20-70 years; 3 [42%] women) were moved from quarantine to a hospital for standard isolation treatment. A total of 20 people who were asymptomatic and who had had direct contact with these individuals were closely observed by health care workers at a nearby hotel. Additionally, 800 individuals considered to be at higher risk were moved from quarantine to home isolation for 14 days. There were no locally acquired cases of COVID-19 with indirect links reported in the Haiyu community from the time that the study began on January 23, 2020, to April 10, 2020.

**Conclusions and Relevance:**

These findings suggest that cooperation among the authorities of multiple sectors allowed for the implementation of preventive measures that were associated with limited community transmission.

## Key Points

**Question:**

Which community preventive measures were implemented in a Chinese city to address the coronavirus disease 2019 (COVID-19) pandemic, and were they associated with limited community transmission?

**Findings:**

This retrospective case series included 7 imported COVID-19 cases and 800 individuals at high risk. After the implementation of community measures, no locally acquired case of COVID-19 with indirect links to confirmed cases was identified in the community.

**Meaning:**

The findings of this study suggest that the implementation of community containment strategies by a multidisciplinary team may limit the community transmission of COVID-19.

## Teaser

This case series study examines measures implemented in a community in China to restrict the spread of coronavirus disease 2019.

## Introduction

A novel coronavirus, designated severe acute respiratory syndrome coronavirus 2 (SARS-CoV-2), has been confirmed as the cause of a new outbreak of pneumonia in Wuhan, China.^1^ Since then, studies^2,3^ have found that coronavirus disease 2019 (COVID-19) is transmitted from human to human and is moderately contagious, compared with severe acute respiratory syndrome and Middle East respiratory syndrome. However, current studies^4,5^ estimate that the incubation period for COVID-19 is 2 to 14 days, with potential asymptomatic transmission, which could lead to the infection of a large number of people. Rapid increases in the numbers of diagnosed and suspected cases have concerned Chinese citizens, even as Chinese authorities have enforced multiple measures, such as imposing a cordon sanitaire (ie, quarantine) and closing public transit.^6^ Therefore, we believe that the most important measure is the early identification and quarantine of individuals infected with SARS-CoV-2. This approach would cut the means of transmission and protect people at high risk in the community, measures that are paramount for elimination of this disease.

As of February 7, 2020, the COVID-19 pandemic had spread throughout the China owing to rapid transmission rates: 351 cases had been confirmed in Shenzhen, a populous city in Guangdong province, China.^7^ Shenzhen is the largest immigrant city in China, with approximately 1 000 000 people who travel to and from Hubei province. Local governmental authorities and representatives from the health bureau held several meetings to develop specific and comprehensive measures, including efforts to trace travel from Hubei province and those who come into close contact with people infected with SARS-CoV-2, as well as efforts to treat individuals at higher risk and those who come into close contact with individuals who are infected. These expert panels also discussed how to provide supplies for people who are isolated at home or in hotels.

The purpose of these measures is to prevent community-dwelling residents from becoming infected with SARS-CoV-2. In this study, we report on a community prevention program for COVID-19 and describe the results of achieved in Haiyu, China, after these measures were first implemented on January 23, 2020.

## Methods

A prevention program for COVID-19 has been conducted in the Haiyu community since January 23, 2020. This case series study reports on the main context of this prevention program and assesses outcomes associated with applying these rigorous containment measures. The study was approved by the ethics committee at the People’s Hospital of Baoan District, Shenzhen. The ethics committee waived the requirement for informed consent because data collection was performed as part of a public health investigation of the COVID-19 pandemic. This study is reported following the Strengthening the Reporting of Observational Studies in Epidemiology (STROBE) reporting guideline. For all confirmed COVID-19 cases, contacts and individuals from Hubei province considered to be at high risk were registered at Haiyu Community Hospital. One of us (H.-E.Z.) has been working in the Haiyu community and collected the relevant data from implementation on January 23, 2020, to April 10, 2020.

### Community Prevention Program

A special multidisciplinary team was established by the city government to investigate the spread of this novel coronavirus infection. The team included a general practitioner, a community manager, and an officer of the public safety bureau, who worked together to strictly, cooperatively, and effectively implement these measures. A flowchart of the community prevention program is presented in the Figure.

**Figure.**
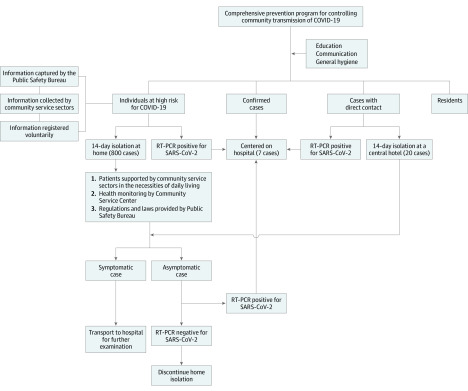
Flow-Chart of the Community Prevention Program COVID-19, coronavirus disease 2019; RT-PCR, reverse transcriptase–polymerase chain reaction; and SARS-CoV-2, severe acute respiratory syndrome coronavirus 2.

To identify individuals from Hubei province who were classified as individuals at high risk, measures were implemented to facilitate the early detection of patients with COVID-19, as well as individuals who might have been exposed to the SARS-CoV-2 but had not yet shown any symptoms.^8^ People in quarantine who were either living or traveling in Hubei province were identified as individuals at high risk. It was determined that such individuals needed to be isolated for a period of 14 days. Local authorities used a database created by the Ministry of Transport, which oversees train, bus, and airline transportation, to develop a system for big data analytics. This system was able to pinpoint cases of suspected exposure and cases at high risk for exposure in Hubei province. Individuals from Hubei province found to be at high risk were asked to scan a Quick Response code that allowed them to register their travel history and symptom record in an online system. In this way, it was possible to trace the contacts of individuals with COVID-19 in Hubei province. The police then shared this information with the community service administration and merged it with local resident demographic information to finalize a list of individuals at high risk. Community health workers made detailed plans to observe these people to prevent community transmission.

Community controls are designed to protect residents from secondary transmission and infection by confirming each person’s identity and checking their temperature. Local authorities also ask the individual about his or her epidemiological links to Wuhan, China, the epicenter of the outbreak. In the case of fever, the community manager registered the information and contacted a general practitioner to send the patient to a medical institution with negative room pressure capacity for further examination. Community administration measures also mandated that every person wear a surgical face mask. Such efforts would help to identify individuals who are potentially infected with SARS-CoV-2 and to prevent nonresidents from entering a given area.

### Prevention Strategy

Individuals with SARS-CoV-2 infection confirmed via reverse transcriptase–polymerase chain reaction (RT-PCR) testing were sent to the Third People’s Hospital of Shenzhen, where they received standard isolation treatment. Anyone who had had direct contact with an individual infected with SARS-CoV-2 had to provide a throat swab sample to test for presence of SARS-CoV-2. Viral RNA was detected by RT-PCR, as described elsewhere.^9^ This test was conducted once the individual was traced by an epidemiologist. If the RT-PCR results were positive for SARS-CoV-2, individuals were required to stay in the hospital; otherwise, individuals were isolated at a central hotel under the control and care of physicians and nurses.

### Isolation and Restriction

Residents from quarantine who did not have direct contact with individuals with COVID-19 were served with mandatory isolation notices and instructed by a health care worker to remain at home for an isolation period of 14 days from the date of registration. It is important to isolate residents who are from epidemic areas but have no signs of illness. We mandated that these residents remain secluded, usually at home, until the 14-day incubation period had ended. At the time of the initial encounter, a team consisting of a general practitioner, a police officer, and a community manager met face-to-face with the individual. They screened each individual’s temperature and evaluated respiratory symptoms. Most importantly, the general practitioner educated individuals about COVID-19, including the mechanisms of COVID-19 transmission, and emphasized the critical importance of general hygiene, particularly hand washing and covering the face with an elbow when coughing. General practitioners also ensured that people with greater exposure were aware of the signs and symptoms of COVID-19 and how to contact their community hospital. Each individual was also asked to provide written informed consent to 14 days of isolation and sign a commitment to obey all regulations and laws. Food and daily necessities were arranged by a community manager. During the incubation period, a general practitioner communicated with individual by telephone and asked them to monitor their health and respiratory symptoms. In the case of fever, the general practitioner called the emergency telephone number for ambulance for a negative air pressure ambulance to transport the individual to the hospital for further examination to decrease community transmission through the use of public transportation.

### Discontinuing Home Isolation

When individuals at high risk finished the 14-day isolation period without self-reported symptoms, a health care practitioner confirmed that the individual was at a very low risk of infection, and a throat swab sample was collected to test for SARS-CoV-2 RNA via RT-PCR. The decision to discontinue home isolation precautions was made by the local health department if negative results were obtained on SARS-CoV-2 RNA RT-PCR testing on the 15th day of home isolation.

## Results

There are approximately 34 686 residents living in Haiyu, including 2382 residents aged 65 years or older. A total of 7 patients with COVID-19 (aged 20-70 years [for anonymity, patient ages are reported by decade only]; 3 [43%] women), all of whom acquired COVID-19 outside the community, were sent to a hospital for standard isolation treatment. Additionally, 20 individuals who were asymptomatic who had been in direct contact with an individual infected with SARS-CoV-2 were placed under close medical observation at a hotel. The Table presents demographic and clinical characteristics of all patients with COVID-19. In addition, 800 people who had returned from Hubei province remained isolated at home for 14 days. After conducting these measures, 800 people finished their home isolation with negative SARS-CoV-2 results on RNA testing via RT-PCR. We found no cluster of locally acquired cases with indirect links to Wuhan in this community, indicating that secondary transmission cases within the community had been significantly limited.

**Table.** Demographic and Baseline Characteristics of Imported Cases Infected With SARS-CoV-2

Characteristic^a^	Case No.						
	1	2	3	4	5	6	7
Admission date	January 24, 2020	February 2, 2020	February 2, 2020	February 3, 2020	February 4, 2020	February 4, 2020	February 4, 2020
Age, y	50s	60s	30s	20s	30	60s	30s
Sex	Man	Man	Man	Woman	Man	Woman	Woman
Epidemiological history^b^	Yes	Yes	Yes	Yes	Yes	Yes	Yes
Direct contacts, No.	8	7	0	0	0	3	2
RT-PCR SARS-CoV-2 results, d of admission							
1	Negative	Positive	Positive	Positive	Positive	Positive	Positive
2	Negative	NA	NA	NA	NA	NA	NA
3	Positive	NA	NA	NA	NA	NA	NA

Abbreviations: NA, not administered; RT-PCR, reverse transcriptase–polymerase chain reaction; SARS-CoV-2, severe acute respiratory syndrome coronavirus 2.

^a^ For anonymity, individuals are identified by number and age is given as decade.

^b^ Classified as yes, had direct contact with an individual infected with SARS-CoV-2 or no, did not have direct contact with an individual with SARS-CoV-2.

## Discussion

At the beginning of 2020, China was experiencing a pandemic crisis, with sharply increasing numbers of cases across the country, especially in Wuhan city.^10^ The Chinese government deployed abundant resources, including health care workers and medical staff from different parts of the country, to fight the COVID-19 epidemic with the severest type of quarantine. The numbers of people infected with SARS-CoV-2 in other Chinese cities, such as Shenzhen and Guangzhou, also showed an upward trend.^10,11^ If local governments cannot contain the original infection sources and cut off secondary or tertiary transmission throughout the community, the virus will spread even farther. Our results show that our community prevention measures were associated with limiting community transmission, a fact that can be attributed to rapid early detection, the isolation of residents, and the implementation of comprehensive multidisciplinary measures. These strategies may prove effective in other areas as well. A study by Güner et al^12^ reported that community prevention measures should be put in place early and adhered to strictly by authorities from multiple sectors to curb the pandemic’s spread. According to recent studies,^3,13^ SARS-CoV-2 has more effective transmission but lower mortality rates than SARS-CoV. However, individuals infected with SARS-CoV-2 are often asymptomatic and highly efficient vectors for transmission of the infection. Governments should therefore promptly carry out rigorous and comprehensive measures to stop the spread of the epidemic. The prevention measures described in this case series may be modified for general application elsewhere.

### Limitations

Our study had some limitations. First, considering the relatively small number of cases imported from Hubei in Haiyu, as well as the relatively short duration of follow-up, the outcomes associated with the intervention measures described in this case series must be assessed with caution. Therefore, more prospective cohort studies about community containment strategies with larger samples should be conducted in the future. In addition, because Shenzhen is a modern Chinese city with a comprehensive management system, these community containment strategies may require modification for successful application in other towns, cities, and countries.

## Conclusions

In this study of the association of community containment with transmission, implementation of rigorous interventions resulted in a total of 0 locally acquired cases of COVID-19 among individuals with indirect links to confirmed COVID-19 cases in Haiyu during the study period. The community prevention measures described limited community transmission.
